# From conceptual domains to operational indicators in palliative end-of-life care: a narrative review

**DOI:** 10.3389/fpubh.2026.1956490

**Published:** 2026-10-13

**Authors:** Catalina Paredes-Coronel, Maria Herrera-Abian, Javier Rocafort-Gil, Angel Jimenez Rodriguez, Jorge Short Apellaniz, Marta Del Olmo, Bernadette Pfang

**Affiliations:** 1 Unidades de Cuidados Paliativos, Unidad de Innovación Clínica y Organizativa, Madrid, Spain; 2 Instituto de Investigacion Sanitaria de la Fundacion Jimenez Diaz, Madrid, Spain; 3 Cátedra de Cuidados Paliativos, Universidad Francisco de Vitoria, Pozuelo de Alarcón, Spain; 4 Jefe de Servicio de las Unidades de Cuidados Paliativos de los Hospitales Públicos de Quirónsalud en Madrid, Madrid, Spain; 5 Hospital Universitario Infanta Elena, Valdemoro, Spain; 6 Hospitales Públicos Quirónsalud (Quirónsalud 4H), Madrid, Spain

**Keywords:** health services research, palliative care, patient-centered care, quality indicators, health care, terminal care

## Abstract

**Background:**

Quality indicators (QIs) are important tools for evaluating and improving care delivery at the end of life. However, despite increasing interest in palliative end-of-life care (PEoLC), there remains limited agreement regarding how quality should be measured, and many proposed indicators remain conceptually rather than operationally defined.

**Objectives:**

To synthesize how quality in PEoLC has been conceptualized in the literature and explore how commonly reported domains may be translated into measurable constructs.

**Methods:**

A narrative (state-of-the-art) review of peer-reviewed literature published between 2015 and 2025 was conducted using MEDLINE (via PubMed), the Cochrane Library, and complementary searches of Google Scholar. Studies addressing quality indicators in PEoLC were identified through a structured search and screened for conceptual relevance. An interpretive synthesis was undertaken to identify recurring domains of quality and explore potential approaches to their operationalization.

**Results:**

Nineteen studies were included. Recurring domains of quality included communication and shared decision-making, symptom management, psychosocial and spiritual support, respect for patient preferences, specialist palliative care involvement, and avoidance of non-beneficial interventions. Although these domains were consistently represented across the literature, they were often described conceptually, with limited attention to how they might be measured in practice. Based on an interpretive synthesis of the evidence, a preliminary framework was developed linking recurrent domains to illustrative examples of potential operational indicators.

**Conclusion:**

While considerable consensus exists regarding the domains that characterize high-quality PEoLC, greater methodological work is needed to translate these concepts into robust and standardized measurement approaches. The framework proposed in this review should be considered exploratory and hypothesis-generating, providing a foundation for future consensus-building and validation studies.

## 1 Introduction

Palliative Care (PC) is a well-established approach aimed at improving quality of life and alleviating suffering among people with serious illnesses (1, 2). Despite its growing integration into healthcare systems worldwide, substantial variation remains in how palliative care services are organized, delivered, and evaluated across settings and countries (3, 4). This variability is partly explained by differences in healthcare systems, institutional models of care, and the fact that, in many countries, palliative care is still not recognized as a distinct medical specialty (4). As a result, the roles, responsibilities, and composition of PC teams often differ considerably, contributing to variation in service provision and potentially affecting the quality and consistency of care received by patients (5, 6). Such heterogeneity highlights the need for robust approaches to evaluating and improving care quality across different contexts.

Within this context, Palliative End-of-Life Care (PEoLC) refers to the application of palliative care principles during the final phase of a life-limiting illness, when care priorities shift toward comfort, symptom relief, shared decision-making, psychosocial support and preparation for death (3, 7). Although PEoLC is conceptually embedded within the broader field of palliative care, the final phase of life presents distinctive clinical, ethical, and organizational challenges (8, 9). Domains such as communication, advance care planning, symptom control, and treatment decision-making are also central to palliative care more generally; however, their urgency, prioritization, and consequences become particularly pronounced as death approaches. PEoLC is distinct from end-of-life (EoL) care as a broad category (which may include aggressive, disease-directed treatments near death) and from hospice care (a service model with setting-specific admission criteria) (10). In PEoLC, palliative strategies are actively integrated within the EoL trajectory to align care with patient values and goals.

Early identification of potential candidates for PEoLC has demonstrated many benefits, including open discussion to clarify treatment preferences and goals of care, improved quality of life and symptom control, lower distress, avoidance of aggressive procedures, lower spending, and potentially longer survival (11). As populations age and the number of people living with advanced chronic illnesses increases, ensuring the quality and consistency of PEoLC has become an important public health priority (12, 13).

Despite growing recognition of its importance, recent international studies have highlighted the continuing challenge of defining and measuring quality in palliative and end-of-life care, as well as the need for robust and standardized indicators to support service evaluation and improvement (14, 15). Quality indicators (QIs) are widely used to evaluate the effectiveness, safety, and patient-centredness of healthcare delivery (16, 17). In palliative and end-of-life care, proposed indicators are commonly derived from domains such as symptom management, communication, advance care planning, psychosocial and spiritual support, and specialist palliative care involvement. However, substantial variation exists regarding which domains should be prioritized, how indicators should be defined, and how they should be measured. Contemporary efforts have focused on developing consensus-based indicators and evaluating the suitability of existing quality measures, although substantial gaps remain in their implementation and standardization (7, 14, 18).

Although key domains of quality in palliative care have been extensively described in previous frameworks and guidelines, less attention has been paid to how these concepts can be translated into measurable indicators suitable for quality assessment and improvement activities (19, 20). Consequently, a gap remains between the identification of quality domains and their practical operationalization. The aim of this review is not to identify new domains of quality, but rather to synthesize recurring domains described in the PEoLC literature and explore how they may be translated into operationally oriented indicators. By explicitly linking conceptual domains to potential measurement approaches, this review proposes a preliminary conceptual framework intended to support future indicator development, consensus-building, and empirical validation efforts in PEoLC.

### 1.1 Objectives

The aim of this narrative (state-of-the-art) review is to synthesize and critically interpret how quality in palliative end-of-life care (PEoLC) has been conceptualized in the literature. Specifically, the review seeks to identify and summarize recurring domains of quality described in PEoLC, recognizing that many of these domains are inconsistently defined and only rarely operationalized as measurable indicators.

Importantly, the objective is not to identify new domains of quality, but rather to explore how established concepts may be translated into operationally oriented measures. By explicitly linking recurrent conceptual domains to potential measurement approaches, this review proposes a preliminary conceptual framework intended to support future quality indicator development, consensus-building, and empirical validation efforts. In doing so, it seeks to address the gap between conceptual descriptions of quality and their practical application in evaluation and quality improvement activities.

To guide this synthesis, the review was structured around the following question: How is quality in palliative end-of-life care conceptualized in the literature, and to what extent can these conceptual domains be translated into measurable and operationally oriented indicators?

## 2 Methods

### 2.1 Design

This study is a narrative (state-of-the-art) review aimed at synthesizing and interpreting how QIs in PEoLC have been conceptualized in the literature. Narrative reviews are particularly suited to areas where concepts remain heterogeneous, terminology is evolving, and the evidence base spans diverse study designs and contexts. Owing to persistent conceptual ambiguity in PEoLC and the lack of standardized QIs, this approach facilitates the integration, interpretation, and critical appraisal of diverse perspectives to address complex conceptual questions. Consistent with the objectives of a narrative review, the intention was not to generate an exhaustive synthesis of all available evidence, but rather to identify and interpret conceptually relevant literature capable of informing a structured understanding of quality in PEoLC (21, 22).

### 2.2 Search strategy

Consistent with recommendations for narrative reviews, a transparent and concept-driven approach to literature identification was employed. In accordance with SANRA (21) recommendations, the search process was explicitly documented to support an interpretive synthesis of quality indicators in palliative end-of-life care (PEoLC).

Searches were conducted between January 1, 2015, and January 1, 2025, in MEDLINE (via PubMed) and the Cochrane library using combinations of controlled vocabulary (e.g., MeSH terms) and free-text keywords related to palliative care, end-of-life care, terminal care, quality indicators, quality measures, and quality assessment. The search strategy was intentionally focused on publications addressing quality indicators or quality measurement frameworks relevant to PEoLC. Google scholar was used as a complementary source to identify additional relevant publications and support citation tracking of key articles. Reference lists of included studies were also manually reviewed to identify further eligible publications.

Retrieved records were managed using reference-management software and screened manually by title, abstract, and full text. Consistent with the narrative nature of the review, study selection was guided by conceptual relevance to the review objectives. Study-selection decisions were discussed among the authors and resolved through consensus.

### 2.3 Eligibility criteria

We included peer-reviewed publications addressing quality indicators (QIs) in palliative end-of-life care (PEoLC), including original research, reviews, qualitative studies, and policy-focused publications. To reflect the relatively recent development of PEoLC as a distinct focus of inquiry, the search was limited to studies published in English between January 1, 2015, and January 1, 2025.

Studies were considered eligible if they addressed the conceptualization, measurement, evaluation, or development of QIs relevant to the final phase of life. To maintain conceptual focus on PEoLC, studies focusing exclusively on broader palliative care quality frameworks were only included when their findings were explicitly applicable to end-of-life care.

We excluded studies focused solely on disease-specific indicators, general palliative care indicators without explicit relevance to end-of-life care, economic evaluations not addressing quality measurement, study protocols, grey literature, and letters to the editor.

Study selection followed a structured but interpretive process, in which titles, abstracts, and full texts were evaluated for their relevance to the objectives of the review. Records were managed using reference-management software, and eligibility decisions were discussed among authors and resolved through consensus.

### 2.4 Selection process

Literature selection followed a structured process based on the conceptual aims of the review. One author performed the initial screening of titles, abstracts, and full texts to identify studies relevant to the objectives of the review. Two senior authors subsequently reviewed the selected articles, assessing their relevance and completeness. Any uncertainties regarding inclusion were discussed among the authors and resolved through consensus.

### 2.5 Data extraction

One author (C. P. C.) extracted descriptive and conceptual information from each study into a structured matrix, including study characteristics, quality indicator labels, operational descriptions, domains, and care settings. Two senior authors (B. P. And M. H. A.) independently reviewed the extracted material to ensure accuracy, completeness, and conceptual coherence with the aims of the review. Any uncertainties or discrepancies were discussed and resolved through author consensus. Extracted data included study objectives, outcomes, perspectives, as well as the definitions, sources, and rationale of the quality indicators described. Given the heterogeneity of study designs and conceptual approaches, an interpretive analytical approach was adopted. Rather than seeking quantitative aggregation, the synthesis focused on identifying recurring concepts, domains, and themes related to quality in PEoLC across different studies. Indicators were subsequently grouped into domains according to similarities in their underlying concepts, intended clinical purpose, and patient-centered focus, with the aim of developing a structured representation of how quality is conceptualized within the literature.

### 2.6 Synthesis method

Given the heterogeneity of the included literature, an interpretive synthesis approach was adopted to identify recurring themes and patterns in the conceptualization of quality indicators within PEoLC. Indicators and quality measures were grouped into domains according to their conceptual focus, clinical purpose, and underlying patient-centered principles. The resulting domain structure was iteratively reviewed by all authors, with modifications discussed until consensus was reached regarding the conceptual boundaries and content of each domain.

A narrative synthesis was then conducted to examine how these domains were described, applied, and measured across different studies and care settings. Particular attention was paid to the relationship between conceptual domains and existing measurement approaches.

Following identification of the recurring quality domains, an interpretive framework-development process was undertaken. Conceptual domains identified in the literature were first synthesized as higher-order constructs and subsequently organized into a preliminary framework intended to facilitate future quality measurement in PEoLC. To enhance transparency, the contribution of individual studies to each domain was mapped during the synthesis process.

Finally, conceptually overlapping indicators and quality measures were compared and consolidated to explore potential approaches to operationalization. The examples of operational indicators presented in the resulting framework were not extracted as validated measures from individual studies. Rather, they were developed by the authors as illustrative examples intended to demonstrate how the literature-derived domains might be translated into measurable constructs for future research and indicator-development initiatives. Accordingly, these examples should be interpreted as heuristic and hypothesis-generating rather than as validated or recommended quality indicators. This approach is consistent with established quality-indicator development methodologies, in which literature-derived conceptual domains constitute the foundation for subsequent indicator specification, consensus development, and validation (23, 24).

### 2.7 Methodological considerations

This narrative review integrates heterogeneous evidence to explore QIs in PEoLC, a field characterized by considerable conceptual variability. Consistent with recommendations for narrative reviews, the approach prioritized conceptual synthesis while maintaining transparency in study identification and selection. The search strategy was concept-driven rather than exhaustive, focusing on literature explicitly addressing QIs in PEoLC, which may have excluded relevant contributions from broader palliative care frameworks or related disciplines.

Given the heterogeneous nature of the included literature, comprising reviews, qualitative studies, observational studies, and policy-oriented publications, no formal quality appraisal was undertaken. Accordingly, the purpose of the review was not to evaluate intervention effectiveness or generate evidence-based recommendations, but rather to identify, synthesize, and organize recurring quality domains within PEoLC. Although study selection and synthesis involved interpretive judgment, discussion and consensus among authors were used to enhance methodological rigor.

Consequently, the identified domains should be interpreted as conceptually derived constructs rather than evidence-weighted categories. Furthermore, the proposed operational indicators were developed as illustrative examples intended to demonstrate how literature-derived domains might be translated into measurable constructs. As such, they represent a preliminary conceptual framework and should be regarded as heuristic and hypothesis-generating rather than as validated quality indicators. Formal consensus procedures, stakeholder involvement, and empirical validation remain necessary before implementation in clinical practice can be recommended.

References published after the search end date were included only during the revision process to strengthen methodological and discussion-related aspects of the manuscript. These publications were not incorporated into the evidence base used for domain identification, synthesis, or framework development.

## 3 Results

### 3.1 Summary of results

Searches across all sources yielded a large number of potentially relevant records, particularly through Google Scholar, requiring progressive screening according to the predefined eligibility criteria. Records were reviewed by title and abstract and assessed for their conceptual relevance to quality indicators in PEoLC. Following this iterative selection process, 26 publications were identified for full-text review, of which 19 were included in the final narrative synthesis.

Excluded studies focused exclusively on broader palliative care indicators without explicit relevance to end-of-life care, disease-specific indicators, economic evaluations unrelated to quality measurement, or provided insufficient information regarding the definition or application of quality indicators.

The final set of included studies comprised a heterogeneous body of literature, including original research, reviews, qualitative studies, and policy-oriented publications. The relatively small number of eligible studies reflects both the limited literature explicitly addressing quality indicators in PEoLC and the continuing conceptual variability surrounding the definition, measurement, and application of quality in this field.

### 3.2 Synthesis into domains and operationalization of QIs

The included studies showed substantial variability in how quality in PEoLC is defined, conceptualized, and measured. Although several recurring domains were consistently identified across the literature, including communication, symptom management, and respect for patient preferences, these domains were frequently described at a conceptual level, with limited agreement regarding their operational definition or measurement.

To explore common patterns across studies, an interpretive synthesis was undertaken. Indicators and quality-related concepts were grouped into domains according to similarities in their underlying purpose, content, and patient-centered focus. This process revealed a recurring disconnect between the conceptual recognition of quality domains and the availability of clearly defined measures suitable for evaluation and quality improvement activities.

To address this observation, recurring domains were linked to examples of potential operational indicators derived from the literature synthesis and informed by existing quality measurement approaches. The resulting framework, presented in Table 1, is intended to illustrate how commonly described domains might be translated into measurable constructs. It should be interpreted as a preliminary conceptual framework rather than a validated set of quality indicators and is intended to support future consensus-building, indicator development, and empirical validation efforts in PEoLC.

**Table 1:** Conceptual domains identified in the literature and their translation into operationally oriented quality indicators.

Domain	Conceptual definition	Example indicator	Potential measurement
Communication and shared decision-making	Timely, clear and empathetic communication with active patient involvement in decision-making	Documentation of goals-of-care discussion	% of patients with documented ACP or goals-of-care discussions
Specialist palliative care (SPC)	Access to and integration of multidisciplinary palliative care expertise	Timing of referral to SPC services	% of patients receiving SPC input before last weeks of life
Physical Symptoms Control	Assessment and control of physical suffering (e.g., pain, discomfort)	Regular symptom assessment	Frequency of symptom scoring using validated tools
Relief of psychological and mental distress	Identification and management of anxiety, depression, and emotional suffering	Screening for psychological distress	% of patients assessed for anxiety/depression
Spiritual support	Addressing spiritual or existential concerns at end of life	Documentation of spiritual needs assessment	% of patients with recorded spiritual care evaluation
Psychosocial support	Addressing social context, caregiver needs, and emotional support	Provision of psychosocial interventions	% of patients/families receiving social or psychological support
Respect for patient and family preferences	Alignment of care with patient values and goals	Advance care planning documentation	Concordance between preferred and actual place of care/death
Appropriate care environment	Delivery of care in a setting aligned with patient needs and preferences	Place of death consistent with preference	% of deaths occurring in preferred setting
Respectful and dignified care	Provision of compassionate, person-centered care	Patient-reported experience of dignity and respect	Patient/family satisfaction measures
Avoidance of non-beneficial interventions	Limitation of interventions that prolong suffering without benefit	ICU admission near end of life	% of ICU admissions in last 30 days of life
Documentation	Recording and transfer of relevant clinical and personal information	Completion of care plans and discharge summaries	% of complete and updated patient records

### 3.3 Conceptual domains and potential quality indicators in PEoLC

For consistency with the aims of this review, all quality-related concepts identified in the literature were interpreted from a PEoLC perspective. Although some studies referred more broadly to palliative care or end-of-life care, their findings were considered relevant when they addressed issues applicable to the final phase of life.

The evidence base was heterogeneous, comprising reviews, qualitative studies, observational research, and policy-oriented publications conducted predominantly in high-income countries and hospital settings. Through the interpretive synthesis, 11 recurring domains of quality in PEoLC were identified based on concepts repeatedly reported across the included studies. These domains were consistently represented across the literature but were often described as guiding principles or desirable aspects of care rather than as clearly defined and measurable quality indicators.

To enhance transparency regarding framework development, Table 2 summarizes the frequency with which each domain was identified across the included studies. While some domains, such as effective communication, physical symptom relief, and specialized palliative care involvement, were reported across a substantial proportion of the literature, others appeared less frequently. The frequency of occurrence should be interpreted as an indicator of recurrence within the literature rather than as a measure of evidentiary strength. The domains are presented below and summarized in Table 1 together with examples of potential measurement approaches derived from the literature synthesis.

**Table 2:** Literature support for the domains included in the proposed PEoLC framework.

Domain	Studies supporting the domain (n)	Key supporting references
Communication and shared decision-making	9	3,4,6,12,17,25–28
Specialist palliative care (SPC)	11	5,6,13,20,25,27–32
Psychosocial support	5	3,4,6,27,30
Spiritual support	5	4,5,7,30,32
Physical Symptoms Control	11	3–5,7,12,13,17,19,30–32
Relief of psychological and mental distress	8	3–5,13,19,20,27,31
Avoidance of non-beneficial interventions	2	31,33
Respectful and dignified care	2	3,30
Respect for patient and family preferences	10	3,6,12,17,19,20,27,28,30,32
Appropriate care environment	5	3,6,20,25,29
Documentation	3	7,17,19

Numbers indicate the frequency with which each domain was identified across the included studies. Domains were retained for framework development when supported by at least one eligible publication and subsequently refined through interpretive synthesis by author consensus.

Several domains showed areas of conceptual overlap, reflecting the inherently multidimensional nature of PEoLC. The framework was not intended to define mutually exclusive categories but rather to identify recurring dimensions of quality that may require separate consideration during quality assessment. Domains such as communication and shared decision-making, respect for patient and family preferences, and documentation are closely interconnected but represent different aspects of quality assessment. Similarly, psychological distress and psychosocial support address related but distinct dimensions of patient and family care.

### 3.4 Analysis of conceptual domains and potential quality indicators used in palliative end-of-life care

For all domains, the proposed operational indicators should be interpreted as illustrative examples of potential measurement approaches rather than validated quality indicators.

#### 3.4.1 Communication and shared decision-making

Communication was one of the most consistently reported domains of quality in PEoLC, appearing in nine of the included studies (3, 4, 6, 12, 17, 25–28).

It encompasses timely, clear, and compassionate exchanges between healthcare professionals, patients, and families regarding prognosis, goals of care, and treatment preferences, and decision-making.

Across the literature, communication is consistently regarded as a cornerstone of high-quality PEoLC. Several studies also highlighted the importance of shared decision-making as a component of communication (26, 29), emphasizing the need to align clinical decisions with patients’ values, preferences, and goals of care. However, both communication and shared decision-making were frequently described as desirable aspects of care rather than as clearly defined and measurable quality indicators.

Examples of measurement approaches reported in the literature include the documentation of goals-of-care discussions, advance care planning conversations, and patient-reported involvement in decision-making (30, 31). These measures may help evaluate the extent to which care is aligned with patients’ preferences and support the delivery of person-centered care during the final phase of life.

#### 3.4.2 Specialist palliative care

Specialist palliative care (SPC) involvement was one of the most frequently identified domains, appearing in 11 of the included studies. SPC teams, typically composed of multidisciplinary professionals such as physicians, nurses, social workers, and spiritual care providers, aim to address the complex physical, psychological, social, and spiritual needs of patients and their families (5).

Across the literature, SPC involvement was consistently associated with improved coordination of care, enhanced symptom management, and better alignment of care with patient preferences (5). Despite its recognized importance, however, the domain was often described conceptually, with limited guidance regarding its measurement or evaluation.

Several studies referred to measures related to the accessibility and integration of SPC services, including the timing of referral, the proportion of patients receiving specialist palliative input during the final phase of life, and the availability of multidisciplinary assessment before death. These measures illustrate potential approaches to operationalizing SPC involvement within PEoLC and may help evaluate the extent to which healthcare systems provide coordinated end-of-life care (32).

#### 3.4.3 Physical symptoms control

Physical symptom management was one of the most frequently reported domains of quality in PEoLC, appearing in 11 of the included studies. It encompasses the assessment and relief of physical suffering, including pain, constipation, dysphagia, and other distressing symptoms that commonly occur during the final phase of life (20).

Across the literature, effective symptom control is consistently regarded as a fundamental component of high-quality PEoLC and a key contributor to patient comfort and dignity. However, several studies also reported persistent variation in symptom assessment and management practices, highlighting ongoing gaps between guideline recommendations and routine care (4, 33, 34).

Examples of measurement approaches described in the literature include the frequency of symptom assessment, the use of validated symptom assessment tools, and the timely provision of appropriate palliative interventions. These measures have been used to evaluate how effectively physical suffering is recognized and managed in clinical practice.

#### 3.4.4 Relief of psychological and mental distress

Relief of psychological and mental distress is one of the most frequently addressed quality indicators in PEoLC. Anxiety and depression emerged as one of the most frequently represented domains in the included literature. Anxiety and depression were consistently identified as important concerns, contributing to increased symptom burden and reduced quality of life (4, 27). Both pharmacological interventions, such as anxiolytics, and non-pharmacological approaches, including psychological and creative therapies, were described as important components of high-quality PEoLC (3). Despite this recognition, the assessment and management of psychological distress were often inconsistently reported across studies.

Several studies referred to measures related to the identification and management of psychological symptoms, including routine screening for anxiety and depression, documentation of psychological support interventions, and access to mental health or supportive care services. These measures illustrate potential approaches to operationalizing psychological care within PEoLC and highlight the importance of structured assessment processes when evaluating subjective experiences such as emotional distress.

#### 3.4.5 Spiritual support

Spiritual support was consistently identified as an important component of quality in PEoLC, reflecting the need to address existential and spiritual concerns that often become more prominent as death approaches (5, 20). Across the reviewed studies, spiritual care was frequently highlighted as a priority for patients and families (4, 7, 19, 20).

Several studies emphasized the importance of proactively exploring spiritual needs, as patients may be reluctant to initiate these discussions themselves (17). Attention to spiritual concerns has also been associated with improved psychological well-being and reduced emotional distress (6, 35).

Examples of measurement approaches reported in the literature include documentation of spiritual assessments, access to spiritual care services, and evidence that identified spiritual needs are incorporated into care planning. These measures may help evaluate the extent to which this dimension of care is addressed within PEoLC.

#### 3.4.6 Psychosocial support

Psychosocial support encompasses the social and relational dimensions of PEoLC, including caregiver burden, family dynamics, social isolation, practical support needs, and preparation for bereavement. Unlike psychological distress, which focuses primarily on emotional symptoms experienced by patients, psychosocial support addresses the wider social context in which care is delivered (17, 20).

Several studies emphasized the contribution of multidisciplinary teams, particularly social workers, in supporting vulnerable patients and coordinating care across settings, especially in community- and home-based services (7, 25). Counseling, caregiver support initiatives, and preparation for bereavement were frequently described as interventions associated with improved emotional outcomes and greater satisfaction with care (3, 35).

Assessment of this domain frequently relied on evidence of psychosocial evaluation, access to social and psychological support services, and documentation of interventions addressing family and caregiver needs, reflecting the extent to which the wider social context of illness is incorporated into care planning.

#### 3.4.7 Respect for patient and family preferences

Respect for patient and family was one of the most frequently represented domains, appearing in 10 of the included studies. This domain reflects the extent to which care is aligned with individual values, goals, and cultural expectations, particularly in situations where patients may no longer be able to express their wishes.

The literature consistently emphasizes the importance of advance care planning, shared decision-making, and family involvement in care planning and communication (17, 20). At the same time, several studies noted that organizational and structural constraints, particularly in acute care settings, may limit meaningful participation by patients and families (6, 13).

Alignment between documented wishes and the care ultimately delivered emerged as a recurring theme across studies, suggesting that the presence and use of advance care planning records may offer valuable insight into preference-concordant care.

#### 3.4.8 Appropriate care environment

The environment in which care is delivered has an important influence on the quality of PEoLC. Although many patients express a preference to remain at home during the final phase of life, hospitals continue to be the most common place of death in many high-income countries (5, 17, 34). This apparent mismatch reflects not only patient preferences but also the availability of community resources, caregiver support, and healthcare infrastructure (3, 27). While hospitals provide immediate clinical expertise, they may be perceived as less personal or comfortable environments (17, 28). Conversely, home-based care may offer greater emotional comfort and autonomy but requires sufficient professional support to ensure safe and effective symptom management.

Measures frequently discussed in relation to this domain included place of death relative to patient preference, availability of home-based services, and continuity of care across settings, all of which provide insight into whether care environments support patient-centered end-of-life care.

#### 3.4.9 Respectful and dignified care

Respectful attention, expressed through compassion, dignity, and personal recognition, presents a fundamental expectation of high-quality PEoLC (34). Although explicitly described in 2 of the included studies it was implicitly present across much of the literature.

Patients value being treated as individuals whose preferences, identities, and personal circumstances were recognized and respected, while families emphasized the importance of emotional support, preparation, and feeling included in the care process (20). These relational aspects often shaped overall perceptions of care quality as strongly as clinical outcomes.

Because respectful attention is inherently interpersonal, assessment tends to rely on patient- and family-reported experiences, documentation of person-centered care practices, and evidence of dignity-preserving approaches throughout the care trajectory.

#### 3.4.10 Avoidance of non-beneficial interventions

Avoidance of non-beneficial interventions (NBIs) emerged as a recurring recommendation across palliative care and PEoLC frameworks (34, 36). NBIs refer to treatments that may prolong life without meaningful benefit or that impose burdens disproportionate to their expected clinical value (36).

Examples described in the literature included intensive care admissions near death, cardiopulmonary resuscitation in patients with limited prognosis, invasive procedures, and continuation of treatments unlikely to improve comfort or quality of life (20, 28, 36). Their occurrence often reflected broader challenges related to prognostic uncertainty, communication, and alignment of treatment decisions with patient goals.

Consequently, patterns of treatment intensity during the final weeks of life, together with the concordance between interventions and documented care goals, may provide useful insight into the appropriateness of care delivered near death.

#### 3.4.11 Documentation

Documentation was consistently identified as an enabling element of high-quality PEoLC, supporting continuity of care, communication across services, legal clarity, and adherence to patient preferences (5, 12, 27).

Several studies emphasized the importance of maintaining accurate and accessible records of care plans, advance directives, and key clinical decisions. Inadequate documentation was associated with fragmented care, difficulties during transitions between settings, and increased risk of interventions that were inconsistent with patient wishes (20). Conversely, well-maintained records facilitated coordination, supported decision-making, and enabled evaluation of care quality (3, 17).

Rather than representing a discrete intervention, documentation functions as a cross-cutting mechanism through which multiple quality domains can be translated into practice and monitored over time. Measures addressing the completeness, accessibility, and continuity of records may therefore provide valuable information regarding overall quality of care.

### 3.5 Synthesis of QI in PEoLC

Taken together, the reviewed literature identified 11 recurring domains of quality in PEoLC (Table 1). These domains informed a preliminary conceptual framework linking key aspects of care to potential approaches for measurement, providing a basis for future consensus-building and validation efforts.

## 4 Discussion

This narrative review synthesizes the literature on quality indicators in PEoLC, highlighting both substantial agreement regarding the domains that characterize high-quality care and persistent gaps in their operationalization (37). Across the reviewed studies, domains such as communication, symptom management, specialist palliative care involvement, psychosocial and spiritual support, and respect for patient preferences were consistently identified as essential components of quality. However, these domains were predominantly described as conceptual principles rather than clearly defined or measurable indicators, limiting their applicability to quality assessment, benchmarking, and service evaluation.

A central finding of this review is the gap between conceptual recognition and practical measurement. While considerable consensus exists regarding what constitutes high-quality PEoLC (38, 39), there remains limited agreement on how these domains should be measured in routine clinical practice (15, 40). This lack of operational clarity restricts the use of QIs for audit, benchmarking, and system-level evaluation, and contributes to variability in care delivery across settings and healthcare systems (7).

To address this gap, this review moves beyond descriptive synthesis by proposing a preliminary framework that links recurrent domains of quality to potential operational indicators. Although previous frameworks have largely focused on identifying and categorizing domains of quality in palliative care (7, 14, 15), less attention has been paid to how these concepts might be translated into measurable constructs (41, 42). The contribution of this review, therefore, lies not in identifying new domains, but in explicitly exploring how established concepts may be operationalized within a PEoLC context. Importantly, the proposed indicators should be regarded as illustrative and exploratory examples rather than a validated set of QIs. Their purpose is to provide a conceptual basis for future indicator development, consensus-building, and empirical validation. Accordingly, the framework should be viewed as a conceptual starting point for future indicator development rather than as a definitive taxonomy of PEoLC quality indicators. An important observation arising from this review is that the identified domains differ substantially in their degree of measurement maturity. Domains such as advance care planning, documentation, and healthcare utilization have been operationalized in previous quality measurement initiatives through relatively specific indicators. In contrast, domains including dignity, spiritual care, and psychosocial support remain conceptually important but have been less consistently translated into measurable indicators within the literature. This variation should not be interpreted as evidence of lesser importance but rather as an indication of areas where further methodological development is needed before robust, reliable, and widely applicable quality indicators can be established. In this regard, the proposed framework may help identify priority areas for future indicator development and validation.

Another important finding is the substantial overlap between broader palliative care and PEoLC domains (8, 43, 44). This overlap was expected and should not be interpreted as evidence that PEoLC lacks conceptual specificity. Rather than representing a separate framework, PEoLC may be understood as a distinct phase within the palliative care continuum in which established domains acquire greater urgency and clinical relevance. The distinction therefore lies less in the domains themselves than in their timing, prioritization, and implications for decision-making. Advance care planning, discussions regarding goals of care, continuity of care, and avoidance of non-beneficial interventions, for example, become particularly important as patients approach the end of life (45–47). These findings suggest that quality assessment in PEoLC may not require entirely different domains from those used in broader palliative care, but rather indicators capable of capturing the specific challenges and priorities associated with the final phase of life.

Beyond these conceptual considerations, the review also highlights the importance of contextual and organizational factors. The predominance of hospital-based studies contrasts with patients’ frequently reported preference for home-based care, suggesting a potential mismatch between service provision and patient priorities (5, 17, 34). In addition, continuity and transitions of care emerged as recurrent themes, reinforcing the importance of coordination across care settings as patients move through the final stages of illness.

### 4.1 Limitations

Several limitations should be considered when interpreting these findings. First, this study was designed as a narrative state-of-the-art review and therefore did not aim to achieve exhaustive evidence retrieval or perform a formal quality appraisal of included studies. Consistent with this methodology, study selection was guided by conceptual relevance to the review objectives and involved an interpretive component, with domains being identified and refined through author consensus. Although this approach is appropriate for narrative and concept-driven reviews, it may have introduced selection bias and may limit reproducibility compared with systematic review methodologies.

Second, the review focused exclusively on literature explicitly addressing quality indicators in PEoLC and may therefore have excluded relevant contributions from broader palliative care quality frameworks or related fields. Given the substantial conceptual overlap between palliative care and PEoLC, some potentially relevant evidence may not have been captured. Nevertheless, this focus was considered necessary to maintain conceptual clarity regarding quality assessment during the final phase of life.

Third, the identified domains reflect the frequency and conceptual relevance of themes reported in the literature rather than the strength of the underlying evidence, as no formal methodological appraisal of included studies was undertaken. In addition, the proposed framework has not undergone formal consensus procedures, stakeholder review, psychometric testing, or empirical validation. Consequently, its practical applicability remains provisional and requires further methodological development before implementation can be recommended.

Finally, the international applicability of the proposed framework may be limited. Most included studies originated from high-income countries with relatively established palliative care services and organizational structures. Although many of the identified domains are likely to represent broadly relevant dimensions of quality in PEoLC, the feasibility, acceptability, and validity of specific operational indicators may vary across healthcare systems. Consequently, further contextual adaptation and validation will be required before the framework can be applied across diverse settings and models of care.

## 5 Conclusion

This review demonstrates that, although there is broad agreement regarding the domains that characterize high-quality PEoLC, these domains are predominantly described as conceptual constructs rather than operational indicators. By synthesizing the available literature and exploring potential pathways for operationalization, the study provides a preliminary framework linking established domains of quality to measurable constructs. The proposed indicators should be interpreted as exploratory and hypothesis-generating rather than validated measures.

Ultimately, improving the quality of PEoLC requires not only agreement on what aspects of care matter most, but also the development of reliable and context-sensitive methods for measuring them. Future research should focus on stakeholder engagement, formal consensus processes, methodological refinement, and empirical validation to determine the feasibility, reliability, and applicability of these indicators across diverse healthcare settings.
