# Supplementary material for: From conceptual domains to operational indicators in palliative end-of-LIFE care: a narrative review
Source: Front Public Health. 2026 Oct 13;14:1956490. doi: 10.3389/fpubh.2026.1956490 (PMC13711033; doi:10.3389/fpubh.2026.1956490)
Supplement: Supplementary file 1 [file Table_1.pdf]

Supplementary Tables

Supplementary Table 1: Summary of key information from the articles included in this review, including study objectives, setting, quality indicators assessed, measurements, and novel perspectives.

REFERENCE	ARTICLE TYPE AND SCOPE (National/ International)	AIM	QI PROPOSED	SOURCE OF QI	ASSESSED ENVIRONMENT (Hospital, Hospice, Home)	OUTCOMES MEASURED	USED TOOLS	NOVEL PERSPECTIVES
**Lind et al. 2015**[1]	National (Swedish) Policies' Review	**To review existing quality indicators** in national Swedish policy documents relevant to palliative and end of life care.	- Communication and shared decision making.<br><br>-Physical Symptoms Control<br><br>- Documentation	National guidelines for cardiology, pulmonary diseases, stroke, diabetes and dementia.	Hospital, home care	Limited use of QI, mainly registration in SRPC.	Presence of protocols Documentation timing	Emphasizes the **importance of integrating palliative indicators** into disease-specific and general care policies. Identifies the **potential of national registers** like the SRPC as tools for QI implementation and monitoring. -Suggests **future QI development** should target underrepresente

								d areas like spiritual, psychological, and social domains.
**Virdun et al. 2015**[2]	Systematic Review International: evidence from Canada, EEUU and UK	**This systematic review** aimed **to ascertain the five most important elements of inpatient end-of-life care as identified by patients with palliative care needs and their families**	- Communication and shared decision making.<br>- Specialist palliative care (SPC)<br><br>-Respect for patient and family preferences.<br>- Appropriate care	Patients and family preferences	Hospital (acute care)	Relative importance of care elements	Ranking, surveys, satisfaction metrics.	First synthesis of what patients and families rank as most important in hospital-based end-of-life care using quantitative data only.<br>Advocates for **hospital system reform** based on these patient-centred domains to improve end-of-life care quality.

			environment					
**Sandsdalen et al. 2015**[3]	Systematic mixed studies review and Quantitative meta-analysis. International: 33 countries (Europe, Asia, North America)	The aim of this study is to **identify preferences for palliative care among patients in the palliative phase of their illness,** by synthesizing existing research.	- Specialist palliative care (SPC)<br>- Appropriate care environment.	Patients' preferences	Home, hospital, hospice, nursing home	Proportions of patient's preferences. Influencing factors: disease type, age, geography, family support, health system	Quantitative measures	Identifies **persistent global gap** between preferred and actual place of death.<br>-Demonstrates influence of **communication, documentation, and service availability** on preference fulfilment.<br>-Suggests that **systematic processes to elicit and act on patient preferences** are critical to improving quality of end-of-life care.

**Blinderman et al. 2015**[4]	National (U.S-based)Narrative Review	The essentials of such care that are presented in this review are intended **to provide both generalists and specialists in fields other than palliative care with a practical, evidence-based approach to alleviating these**	- Communication and shared decision making.<br><br>-Physical Symptoms Control.<br><br>-Relief of psychological and mental distress<br>-Spiritual support<br>-Psychosocial support	Scientific literature in general	Hospital (acute care)	Consensus-based practice guidelines	Dosing tables, symptom severity scales, symptom management algorithms.	Bridges the **gap between PC specialist expertise and generalist reality**, providing tools for non-PC clinicians. Clarifies misconceptions around "comfort care" as passive or minimalistic by detailing **proactive, expert-level interventions**.

		**symptoms in patients who are dying in a hospital**.						
**You et al. 2015**[5]	Multicentre survey National (Canada)	To determine, from the perspective of hospital-based clinicians, (1) barriers impeding communication and decision making about goals of care with seriously ill hospitalized	- Communication and shared decision making.	Clinicians Surveys to patients and families	Hospital (acute and inpatient care)	Barriers in PC	Multicenter survey	Clinician-perceived **barriers to goals of care discussions** in hospital settings. Offers evidence for developing **multifaceted interventions** that include communication training, decision aids, and cultural change in hospital systems.

		patients and their families and (2) their own willingness and the acceptability for other clinicians to engage in this process.						
**Singer et al. 2016[6]**	Systematic Review. International (U.S, Western Europe, Canada, Australia, New Zeeland and Israel)	To inform how payers and providers should identify patients with "advanced illness" and the specific interventions they should	- Communication and shared decision making.<br>- Specialist palliative care (SPC)<br><br>-Relief of psycholo	Trials outcomes: patients and caregivers, social workers and nurses	Home, Hospital, Outpatient clinics	Pain, Depression, Communication, Satisfaction, Cost, Healthcare use	Qualitative synthesis.	Demonstrates that **nurses and social workers** are pivotal; team-based and home-based interventions are most effective. Identifies strong evidence for **communication, psychosocial outcomes, and patient/caregiver satisfaction**, but **limited**

		implement, we reviewed the evidence to identify (1) individuals appropriate for palliative care and (2) elements of health service interventions (personnel involved, use of multidisciplinary teams, and settings of care) effective	gical and mental distress<br>-Psychosocial support<br>-Respect for patient and family preferences					**evidence** for reducing **overall healthcare costs**<br>Provides clear **guidance for service design**: who to target, which components to include, and expected outcomes.

		in achieving better outcomes for patients, caregivers, and the healthcare system.						
**Cardona-Morrell et al. 2016**[7]	Systematic Review International: 10 countries (U.S., Canada, Australia, South Korea, Brazil, Taiwan, Europe)	To investigate the extent of objective 'non-beneficial treatments (NBTs)' (too much) anytime in the last 6 months of life in routine hospital care.	- Avoidance of non beneficial interventions	English publications	Hospital (acute care, ICU, oncology, general wards)	NBT: ICU admissions, CPR, Chemotherapy, futile meds, invasive procedures, imaging/tests)	Meta-analyses Scores applied to assess quality of included studies	First review to compile a comprehensive, **quantitative taxonomy of measurable indicators** for non-beneficial treatment (NBT) at end of life. -Highlights systemic and clinician-level contributors (e.g., poor communication, lack of documentation, cultural pressures).

								-Argues for the **operationalization of NBT metrics** to inform hospital policy, resource allocation, and ethical care practices.
**Meier et al. 2016[8]**	Literature Review International: U.S., UK, Japan, Netherlands, Israel, Thailand, Iran, Sweden	The authors conducted a literature search for published, English-language, peer-reviewed reports of qualitative and quantitative studies that provided	-Specialist palliative care (SPC) -Physical Symptoms Control -Relief of psychological and mental distress -Spiritual support -Psychoso	Published literature	Home, hospice, hospital	11 core themes and subthemes.	hematic frequency across stakeholder groups (patients, family, HCPs)	First comprehensive **empirical synthesis of stakeholder-defined components of a "good death."** -Calls for **dialogue-based approaches** and **patient-centred tools** to individualize care at the end of life. -Provides foundation for developing **standardized tools and policy**

		a definition of a good death.	cial support<br>-Respect for patient and family preferences<br><br>-Respectful and dignified care					**guidance** around definitions and metrics of a good death.
**Gaertner et al. 2017**[9]	Systematic review and Metanalysis. International: U.S., Canada, UK, Australia, Ireland, Germany	To assess the **effect of specialist palliative care on quality of life** and additional outcome	-Specialist palliative care (SPC)<br>-Physical Symptoms Control.	Published literature: randomised controlled trials with adult inpatients or outpatien	Hospital, Hospice, Community (including home-based care)	Primary outcome patient's' quality of life.<br>Secondary outcomes included symptom burden (pain, fatigue, nausea, and dyspnoea), psychosocial variables (distress, depression,	-Quality Scales<br><br>-Tools that covered at least two dimensions of quality of life (physical,	-Demonstrates that **SPC improves QoL modestly**, especially when **initiated early** and tailored to **unmet needs**, not diagnosis alone.<br>-Offers evidence to shift from **diagnosis-**

		s relevant to patients in those with advanced illness.	-Relief of psychological and mental distress<br>-Avoid NBT	ts treated in hospital, hospice or community settings		anxiety, spiritual wellbeing, social wellbeing, and satisfaction), survival time, place of death, cost of care, and attrition (or completion rate).	psychological or social).	**based referral** to **needs-based screening** for SPC.<br>-Argues against universal SPC and recommends **resource-targeted, integrated models** that support generalist palliative care and escalate when needed.
**Higginson et al. 2017**[10]	Cohort study. International: UK, Dublin, USA	**to determine and compare, across three countries, factors associated with preferences for**	-<br>Specialist palliative care (SPC)<br><br>-Respect for patient and family	Data obtained from patients participating in the cohort	Hospitalary (PC teams)<br>Outpatients' clinics<br>Hospice	Patient's preferences for place of death, treatment goals, associated social/clinical factors	face-to-face interviews (preferences, POS scores, Barthel Index, SOMCT).	Highlights that **preferences and actual place of death often mismatch—**only 23% of those who preferred home died there.<br>- Finds **diagnosis (cancer vs. non-cancer), living**

		**place of death and treatment**, and actual site of death.	preferences<br>- Appropriate care environment					**status, functional ability**, and **treatment goal orientation** influence preference and actual place of death.<br>-Challenges using "home death" as a universal QI; proposes **better QIs would contrast preferred vs. actual location and measure quality at place of death.**
**Pask et al. 2018[11]**	In-depth qualitative interviews, analysed using Framework analysis. National: UK	To explore palliative care stakeholders' views on what makes a	-Effective Communication.<br><br>-Expert care.<br>- Respecting family	Results of Interviews	Hospital, hospice and community	Results of interviews to patients, family, professionals, managers and senior leads.	Developed a conceptual model that includes:<br>- Microsystem	-Highlights that complexity is **dynamic**, not static — care models and resources must be adaptable.<br>-Suggests practical

		patient more or less complex and insights on capturing complexity at patient-level.	and patient preferences,<br>- Psychosocial support,<br>- Appropriate environment.				(individual needs and traits)<br>- Mesosystem (family and provider interactions)<br>- Exosystem (health systems/ services)<br>- Macrosystem (societal values, stigma)<br>- Chronosystem (changes over time)	implications for **triaging patients**, **resource allocation**, and refining the definition of who needs SPC.<br>-Identifies **gaps in current classification systems** that over-rely on diagnosis or physical status.

**Pivodic et al. 2018**[12]	Epidemiological study. International: six European countries: Belgium, Finland, Italy, Netherlands, Poland, England	**To determine the quality of dying and end-of-life** care of nursing home residents in six European countries. Since it is the most common place of dying	-Expert care.<br>-Physical symptoms management<br>-Relief of psychological and mental distress	Results of epidemiological interviews	Nursing homes	Results of an epidemiological survey to staff: nurses or care assistants	Two validated instruments:<br>**EOLD-CAD** (Comfort Assessment While Dying): physical/ emotional distress and well-being in the last week of life<br>**QoD-LTC** (Quality of Dying in Long-Term Care): personhood, closure, preparatory tasks in the last	-Shows that even countries with high palliative care development (e.g., UK, Netherlands) show **deficiencies in symptom relief and care planning**.<br>-Provides **benchmark data** on EOL care quality across Europe and identifies where systemic improvements are needed.<br>- Demonstrates that **shorter length of stay**, **dementia**, and **low functional status** are associated with poorer outcomes—relevant for

							month of life	tailoring QIs and interventions.
**Ahluwalia et al. 2018**[13]	Systematic Review. National (U.S. based)	The purpose of this review was to provide a synthesis of the evidence in palliative care to inform the fourth edition of the National Consensus Project Clinical Practice Guidelines for Quality Palliative Care.	-Expert care<br>-Physical symptoms management<br>-Spiritual support<br>-Relief of psychological and mental distress	Outcomes of selected articles	Hospital, hospice, home, Nursing home	**outcome-based indicators**: symptom burden, QoL, hospital use, satisfaction, spiritual well-being.	empirical studies, quality improvement projects, and randomized trials.	-Supports **integration of QIs into national policy**, clinical practice, and research prioritization.<br>-Serves as a blueprint for **standardized implementation and evaluation** of high-quality palliative care across settings.

**Virdun et al. 2020**[14]	An exploratory qualitative study using semi-structured interviews. National: Australia	To seek the perspectives of Australian patients living with palliative care needs about their recent hospitalisation experiences to determine the relevance of domains noted internationally to be important for optimal	-Effective Communication<br>-Expert care,<br><br>-<br>Respecting family and patient preferences	Answers from interviews	Hospital	Domains previously identified as important for optimal hospital palliative care,	**Exploratory qualitative study**: semi-structured telephone interviews with 21 patients	**Adds two new domains** to the international literature:<br>**-Nutritional needs –** importance of food in wellbeing, appetite, dignity, and comfort.<br>**-Access to clinical specialists** – particularly **nurse specialists**, seen as key to continuity, reassurance, and informed care.<br>Offers patient-derived **recommendations for practice** and **policy reform**, centred on what patients define as quality care.

		inpatient palliative care and how these can be operationalised.						
**Sjöberg et al. 2021**[15]	Retrospective Review in which based on records info about QI are extracted. National: Sweden	The aim of this study was to explore, in the context of Swedish specialised palliative care, the content of documentation in older people's patient records.	-Physical symptoms management<br><br>-Relief of psychological and mental distress<br>-Respecting family and patient preferences,<br><br>-Documentation.	Patient`s records	Specialised home palliative care	Descriptive statistical analysis with inter-rater reliability verification (97%).	**Retrospective review** of 92 deceased older patients' records (n = 22,640 clinical notes). Use of a structured review template with 33 categories and 264 subcategories (e.g., pain,	Highlights a **major gap between holistic palliative care ideals and documentation practices**, especially in psychological, spiritual, and existential areas.<br>-Documents that even in **highly developed SPC systems**, care documentation is still **largely focused on physical symptoms**, particularly pain.

		Research questions: What problems, wishes, aspects of wellbeing, assessment tools, and interventions are documented in patient records and to what extent? What is documented in association with the patients' deaths and to					preferences, assessments)	-Reveals **lack of follow-up on assessment tools**, poor capture of patient wishes regarding companionship at death and spiritual preferences.<br>-Calls for the adoption of **structured, person-centred documentation models** (e.g., the 6 S model) to capture full scope of patient needs and experiences

		what extent?						
**Choudhary et al. 2021**[16]	Narrative Review. National: India	This review aims to recognize the role of palliative care at the end of life.	-Effective Communication<br><br>-Physical symptoms management<br><br>-Relief of psychological and mental distress<br>-Respecting family and patient preferences,<br>-Psychosocial support,<br>-Appropri	Published literature	Hospital, hospice, care home and patient` s fomes	**Narrative review** synthesizing literature, WHO guidance, and empirical studies.	but draws from international reviews,	-Highlights the importance of **early integration** of palliative care and the multidisciplinary team in shaping outcomes.<br>-Advocates for **QoL and place-of-death** as valid quality indicators, even in low-resource settings.<br>-Makes a compelling case for **palliative care as a cost-reducing, QoL-enhancing intervention** at the end of life.

			ate environment, - Respectful attention,					
**Williams et al. 2022**[17]	Results from a collaborative expert workshop. National: Canada	The need for palliative care (PC) will continue to increase in Canada with population aging. Many older adults prefer to “age in place” and receive care in their own	-Expert care, -Physical symptoms management -Spiritual support - Respecting family and patient preferences,<br><br>- Respectful attention,	Results from expert meeting	Community and home-based palliative care	**Domains for future QI development** using interRAI PC as a future data source.	**Qualitative expert workshop** with three structured brainstorming sessions. -Data collected via flipcharts and sticky-note clustering; themes grouped per **National**	-First expert-led consensus in Canada explicitly aimed at developing a **standardized QI set for community-based palliative care**. -Introduced **two home-specific QI domains**: **1.Spiritual care** (patient and family access) **2.Home as the preferred place of death** -Strong emphasis on **early PC integration**,

		homes. Currently, there is a lack of standardized quality indicators (QIs) for PC delivered in the community in Canada.					**Consensus Project (NCP)** domains.	**caregiver support**, and **standardized documentation tools** (interRAI).<br>-Lays the groundwork for the **development of operationalized QIs** from existing health system data.
**Efstathiou et al. 2024**[18]	A modified Delphi technique approach was used with palliative care stakeholders.<br>National: UK	Identifying research priorities is very important for palliative and end-of-life care to ensure research is focused on	-Effective Communication<br><br>-Respecting family and patient preferences.<br>-Physical symptoms management	Results from Delphi-based consensus	Hospital, Hospice and social care systems		**Three-round modified Delphi study**<br>-Round 1: Rapid interviews (n=56)<br>-Round 2: Online Likert-scale survey (n=30)	-Emphasizes **equity** with 3 out of top 7 research priorities focusing on **underserved groups** (ethnic minorities, prisoners, LGBTQ+, etc.).<br>-Identifies **training gaps for generalist staff** – a scalable intervention area.

		evidence gaps. This project aimed to identify and prioritise palliative and end-of-life care research areas within the West Midlands region in United Kingdom (UK).					-Round 3: Ranking of priority items (n=45)	-Reinforces **multidisciplinary care** and patient-centred systems as key levers for quality improvement
**Allard et al. 2024**	Qualitative Study. National: Canada	To propose measures to quantify and conclude reliable indicators	-Physical symptoms management<br>-Spiritual support<br>-Relief of psychological and	Current administrative policies	Home, Hospital (Long-term-care), Palliative care homes.	**Eight administrative indicators (2002–2016)** (e.g., ER visits, ICU use, hospitalizations, place of death	**Qualitative interviews (n = 9)** explored expert interpretation and recommendations.	-Highlights significant **gaps between current administrative indicators and actual care quality**.<br>-Recommends creation of a

			mental distress<br>-<br>Documentation.					**national PEoLC observatory** to improve indicator governance, transparency, and comprehensiveness.<br>-Calls for better **early identification**, **professional training**, and **interdisciplinary integration**, especially in home care settings.
